# Double-Loop Learning in Adaptive Management: The Need, the Challenge, and the Opportunity

**DOI:** 10.1007/s00267-018-1107-5

**Published:** 2018-09-29

**Authors:** Byron K. Williams, Eleanor D. Brown

**Affiliations:** 1 The Wildlife Society, 425 Barlow Place, Suite 200, Bethesda, MD 20814 USA; 2 Science and Decisions Center, U.S. Geological Survey, 12201 Sunrise Valley Drive, Reston, VA 20192 USA; 3 Present Address: Science and Decisions Center, U.S. Geological Survey, 12201 Sunrise Valley Drive, Reston, VA 20192 USA

**Keywords:** Adaptive management, Decision elements, Double-loop learning, Technical and institutional learning, Uncertainty

## Abstract

Adaptive management addresses uncertainty about the processes influencing resource dynamics, as well as the elements of decision making itself. The use of management to reduce both kinds of uncertainty is known as double-loop learning. Though much work has been done on the theory and procedures to address structural uncertainty, there has been less progress in developing an explicit approach for institutional learning about decision elements. Our objective is to describe evidence-based learning about the decision elements, as a complement to the formal “learning by doing” framework for reducing structural uncertainties. Adaptive management is described as a multi-phase approach to management and learning, with a set-up phase of identifying stakeholders, objectives, and other decision elements; an iterative phase that uses these elements in an ongoing cycle of technical learning about system structure and management impacts; and an institutional learning phase involving the periodic reconsideration of the decision elements. We describe a framework for institutional learning that is complementary to that of technical learning, including uncertainty metrics, propagation of change, and mechanisms and consequences of change over time. Operational issues include ways to recognize when the decision elements should be revisited, which elements should be adjusted, and how alternatives can be identified and incorporated based on experience and management performance. We discuss the application of this framework in decision making for renewable natural resources. As important as it is to learn about the processes driving resource dynamics, learning about the elements of the decision architecture is equally, if not more, important.

## Introduction

Adaptive management of natural resources recognizes uncertainty and seeks to reduce or eliminate it through management itself (Nichols and Williams 2012). Its history in natural resources stretches back at least four decades to the work of Holling (1978) and Walters and Hilborn (1978), who first used the phrase “adaptive resources management.” Walters (1986) described adaptive management in terms of the “dual control” problem in engineering (Stengel 1994), whereby learning about a managed system occurs simultaneously with its management. Adaptive management in renewable natural resources involves iterative decision making, the propagation of uncertainty, and the use of management to reduce uncertainty while pursuing other management objectives. In simple terms, it can be described as learning by doing, and adapting based on what’s learned. The overall idea is that managed resource systems are seldom if ever fully understood—i.e., uncertainty exists—and this lack of understanding limits management effectiveness. Thus, it makes sense to account for uncertainty in decision making over time, so as to track the consequences of decisions and adjust management as learning occurs.

The term “uncertainty” in this context means a general lack of predictability about future conditions, with a special focus on uncertain system dynamics and their responses to management. Uncertainty can be thought of as a mirror image of understanding, in that a reduced level of uncertainty corresponds to an increase in understanding. These terms are linked in turn to management performance, in that better understanding of a system being managed can lead naturally to an improvement in management performance. Two categories of uncertainty are emphasized: (1) structural uncertainty, i.e., uncertainty about the structure of the resource system and the processes (such as survivorship and reproduction) that influence its dynamics; and (2) institutional uncertainty, which concerns the elements and architecture of the decision-making cycle itself.

**Fig. 1:**
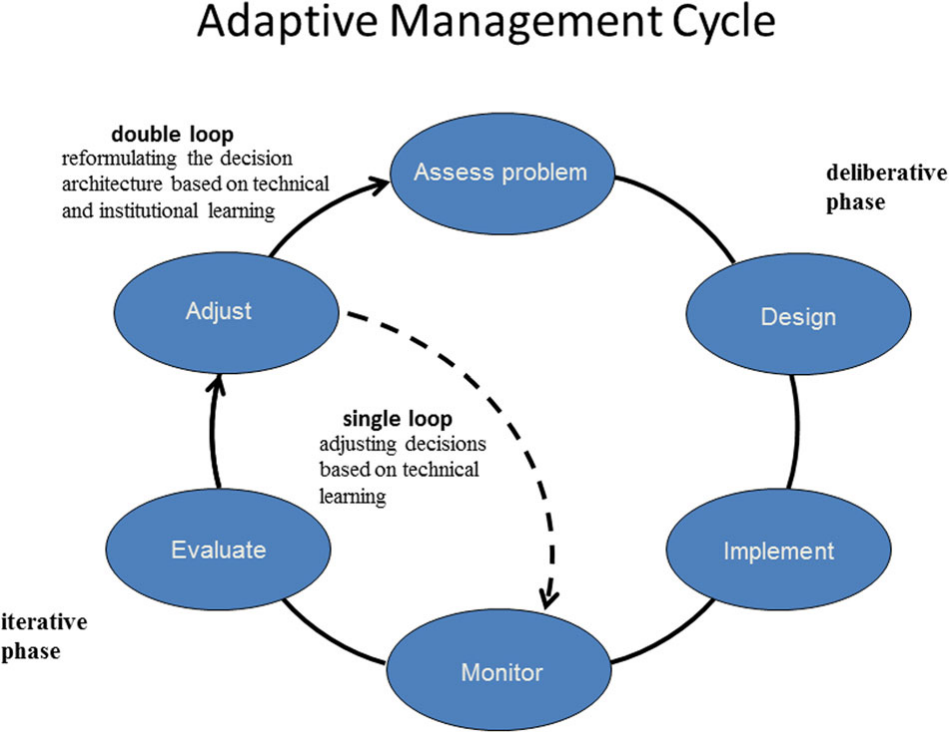
Adaptive management displayed as a cycle with double-loop learning. A deliberative phase includes problem assessment, design of the decision architecture, and implementation. An iterative phase includes monitoring, evaluation of monitoring results, and adjustment of management strategy (from Williams and Brown 2014)

Early on, the emphasis of adaptive management was on the reduction of structural uncertainties about the resource system through repeated sequences of decision making, monitoring, learning, and strategy adjustment (Walters 1986), in a cycle of technical learning. However, current descriptions of adaptive management are usually more inclusive, with technical learning subsumed in a more comprehensive process that also allows for institutional learning and adaptation of the decision-making elements (Fig. 1). Both structural and institutional uncertainties are incorporated in an overall decision-making cycle, with technical learning (sometimes called “single-loop learning” [Argyris and Shön 1978]) represented as a sub-loop in the larger sequence of planning, design, and management (often referred to as “double-loop learning” [Argyris and Shön 1978]). Recent innovation includes “triple-loop learning,” a third level of learning related to reconsideration of underlying values and beliefs, which has become increasingly important in resource governance discussions (Pahl-Wostl 2009). Consideration of uncertainty arising from social processes (Tyre and Michaels 2011) has followed from the recognition that various forms of social learning are critical for developing adaptive management of complex systems.

Although a great deal of work has been done on the theory and operational details of technical learning in adaptive management, there are surprisingly few examples in the literature of an explicit approach for institutional learning. Because managers frequently make changes in decision-making elements as resources are managed through time, there is a need to replace the ad hoc nature of institutional adjustments with a more systematic treatment. Thus, our objective in this article is to provide a framework for evidence-based learning to reduce uncertainties related to the institutional elements of adaptive management. Such a framework complements the formal “learning by doing” formulation for reducing structural uncertainties. Rather than presenting a comprehensive literature review, we highlight a few of many approaches and examples, recognizing the possibility of others depending on the nature of the problem at hand.

In the following sections, we describe the cycle of adaptive management and its relation to uncertainty; illustrate the complete adaptive management cycle with a comprehensive example from the regulation of waterfowl hunting; discuss issues and provide examples relevant to adjustment of each decision element; and consider ways in which uncertainty can be reduced through institutional learning. We discuss adaptive management in the context of the dynamism of biological resources, in which processes such as survivorship and recruitment produce outcomes like harvest yields and changes in population status, and management actions are taken pursuant to intended consequences.

## The Adaptive Management Cycle

**Fig. 2:**
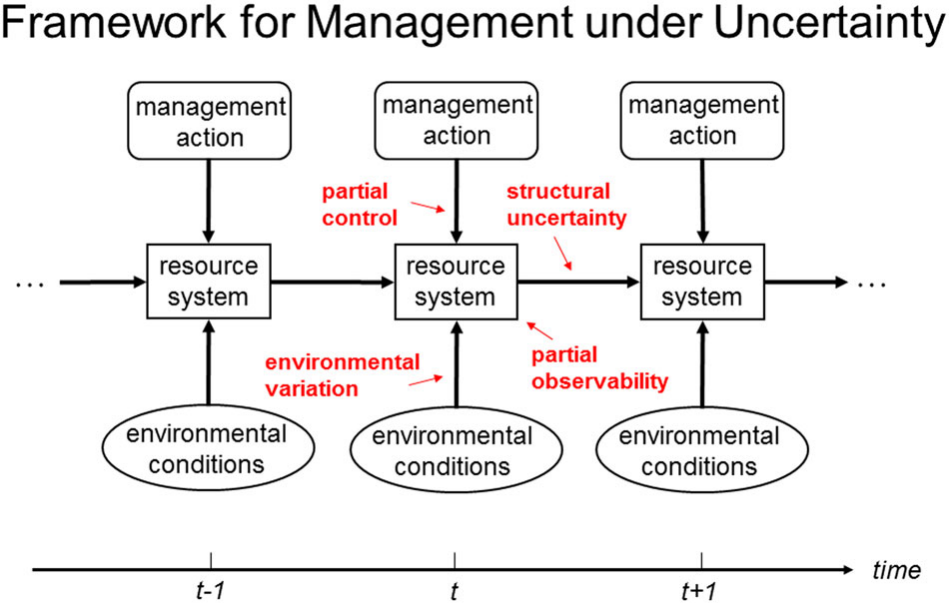
Dynamic resource system, with changes influenced by fluctuating environmental conditions and management actions. Uncertainty factors include partial control, which limits the influence of management actions; environmental variation, which affects resource system status and dynamics; partial observability, which limits the recognition of system status; and structural uncertainty, which limits the ability to characterize system change (from Williams and Brown 2016)

Adaptive management of natural resources is almost always characterized in terms of a resource system’s uncertain responses to management (Williams 2011). A working definition is “decision making that accounts for what is known and what is uncertain about resource dynamics, and seeks to reduce uncertainty so as to improve management over time.” Although varied, discussions of adaptive management usually share common features (Williams and Brown 2014) that include: (1) system changes in response to fluctuating environmental conditions and management actions; (2) environmental variation that induces stochasticity in biological and ecological processes, leading to unpredictable system behaviors; (3) periodic and potentially varying management interventions that can influence system behaviors either directly or indirectly; and (4) limitations on effective management because of uncertainty about the resource system and how it responds (Fig. 2). The fact that management, environmental variation, and system status are dynamic provides an opportunity to improve management by learning.

**Fig. 3:**
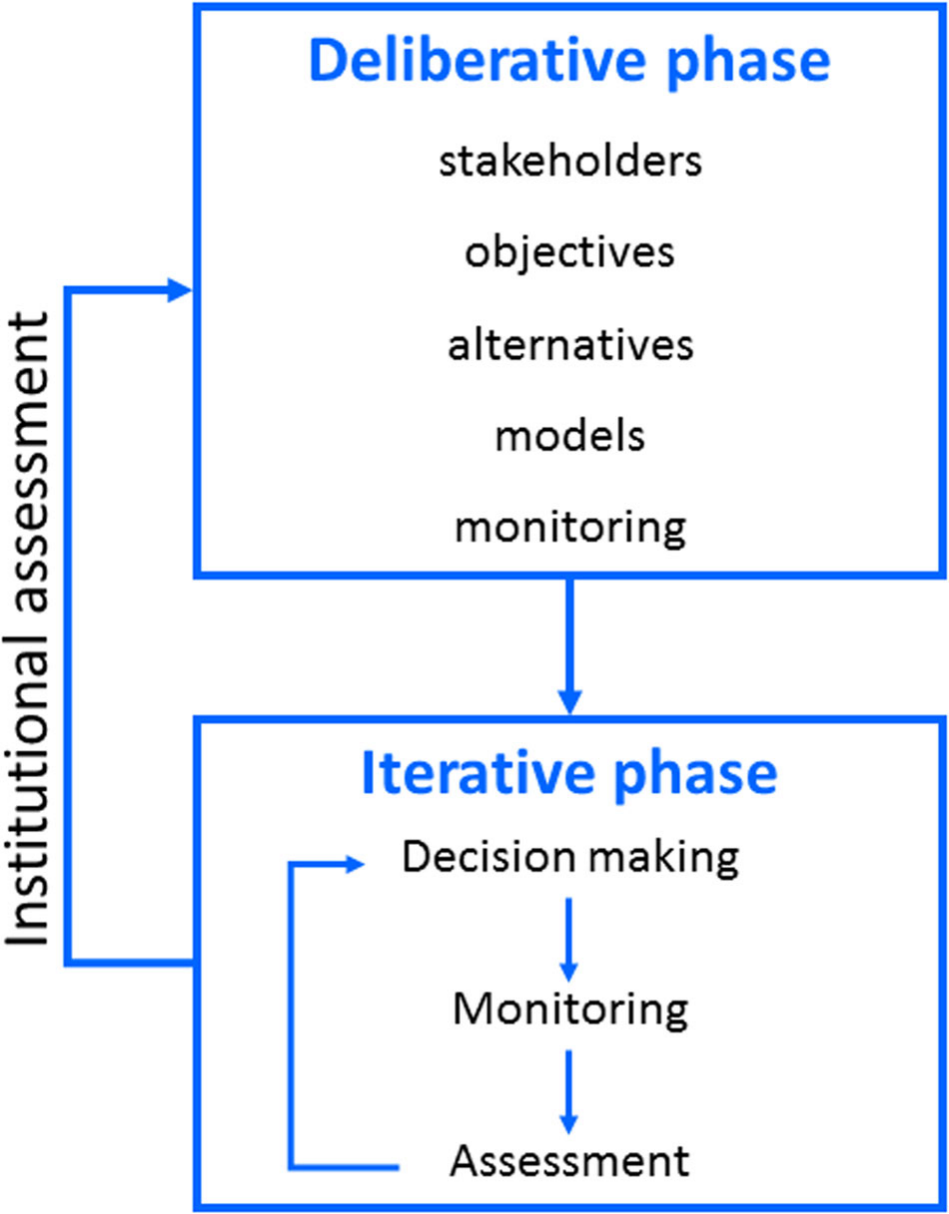
Learning in adaptive management. Technical learning involves an iterative sequence of decision making, monitoring, assessment, and feedback of what is learned into decision making. Institutional learning involves periodic reconsideration of the components in decision making (from Williams and Brown 2014)

A framework for adaptive decision making can be characterized as a process with multiple phases (Williams and Brown 2014) (Fig. 3). A deliberative phase involves framing the resource management issue in terms of stakeholders, objectives, management alternatives, predictive models (including measures of the confidence one places in them), and monitoring protocols. An iterative phase uses these elements in an ongoing cycle of technical learning about system structure, function, and management impacts. Finally, an institutional learning phase focuses on the decision components themselves, by periodically interrupting the iterative cycle of technical learning to reconsider project objectives, management alternatives, stakeholder engagement, and other elements of the deliberative phase (Fig. 3). The institutional learning cycle complements, but obviously differs from, the embedded cycle of technical learning.

It is the iterative application of this framework over time that allows learning to occur. A particular focus in adaptive management is on structural uncertainty, specifically, a lack of understanding (or lack of agreement) about the processes of resource system dynamics. Differing views about how natural processes work and how they respond to management can be framed as hypotheses, captured in predictive models, and investigated through comparisons of predictions to data (Williams and Brown 2012). Predicted consequences of management actions at each decision point can be compared with data produced by monitoring, to determine the relative effectiveness of each of the alternative models in predicting decision outcomes. These comparisons can be used in turn to increase or decrease the confidence one assigns to the models, and in this way the best hypothesis about resource dynamics becomes evident over time. The gradual increase in confidence, leading to identification of the appropriate model, is a key metric for technical learning and improved management. In like manner, the decision elements themselves can be evaluated periodically against observed management performance. Institutional learning and adaptation require revisitation of the decision-making components, and ideally include a “weight of evidence” assessment of the components against criteria for change, along with modification when change criteria are met.

In the next section, we use adaptive management of waterfowl hunting as a comprehensive example to illustrate the full cycle of adaptive management.

## Example of a Complete Cycle of Adaptive Management: Sport Hunting of North American Waterfowl

“Adaptive harvest management” is currently being used for the regulation of sport hunting of waterfowl in North America (Williams 2006; Nichols et al. 2007; Johnson et al. 2015). The approach is based on yearly fluctuations of waterfowl populations, and is adapted to that periodicity. Management involves the annual adjustment of hunting regulations in late summer, in order to influence the size of the harvest during the subsequent fall and winter hunting season. The choice of regulations is based on three factors: waterfowl population status (size and reproduction) in the spring and summer each year; water conditions (number of wetlands) on the prairies in the spring each year; and understanding of (or conversely, uncertainty about) biological processes (reproduction and mortality) that influence population changes from year to year.

**Fig. 4:**
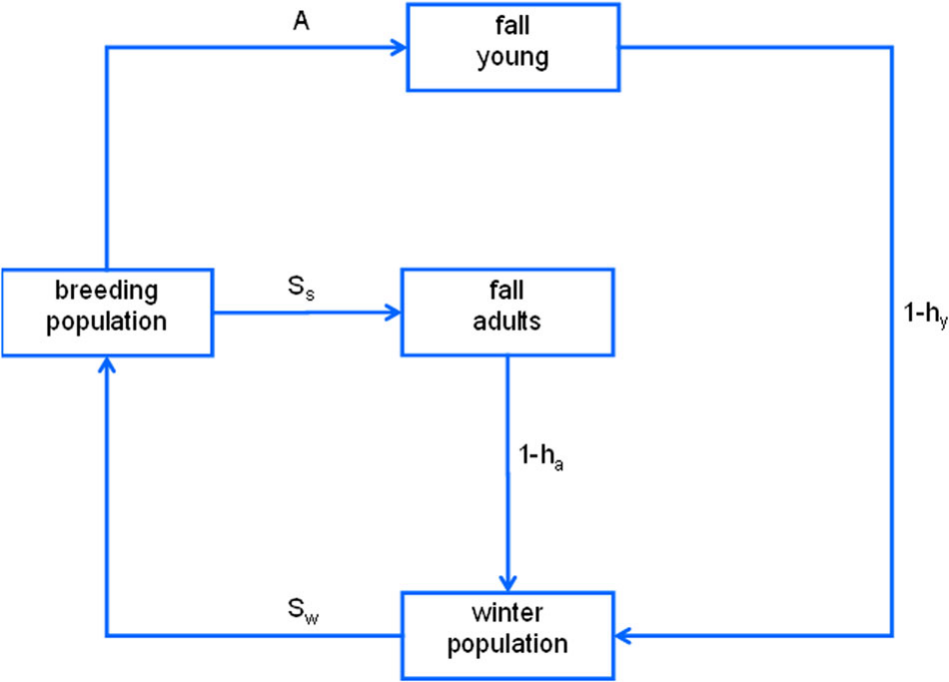
Conceptual model of annual cycle of mallard population dynamics. Model includes survival rates for spring–summer (*S*_*s*_) and fall–winter (*S*_*w*_), along with harvest rates for young (*h*_*y*_) and adults (*h*_*a*_) and age ratio (*A*) for reproduction/recruitment (from Williams and Brown 2012)

Population models in adaptive harvest management are used to represent potential associations among fall harvest, seasonal survivorship, and spring reproduction (Fig. 4). Contrasting hypotheses about the impact of harvest on annual survivorship are incorporated in these models, along with contrasting hypotheses about the importance of density dependence in recruitment. In combination these hypotheses define four different models, each with its own predictions about harvest impacts and each with its own measure of confidence that evolves over time. Every year the US Fish and Wildlife Service establishes flyway-specific “framework” regulations for waterfowl hunting that include the earliest and latest dates for hunting seasons, the maximum number of days in the season, and daily bag and possession limits. Currently, three regulatory frameworks along with a closed season constitute the alternative management actions that are available for setting duck-hunting seasons (Johnson 2011). The participation of stakeholders, an essential component of the regulatory process, is facilitated through an institutional apparatus for public comment and participation in rule making (Johnson 2011).

Adaptive harvest management builds on the predicted consequences of the regulatory alternatives, through comparison with monitoring results (Martin et al. 1979; Smith et al. 1989). The greater a model’s predictive ability, the more heavily it is weighted, and hence the greater role it has in determining regulatory choices. The learning that results from comparing predictions with actual monitoring data is codified by a quantitative Bayesian process of updating the model weights, which are used in determining harvest regulations the following year. It is the propagation of uncertainty over time that allows adaptive harvest management to promote understanding while simultaneously targeting management objectives.

As with the sequential management of most natural resources, the decision-making elements of the hunting regulation process (objectives, management alternatives, monitoring protocols, etc.) are reconsidered over time (e.g., see Johnson et al. 2016). One example is the periodic re-examination of objectives (Johnson 2011) to decide whether revision is needed, perhaps due to management performance or changes in stakeholders’ attitudes. In particular, objectives have been revised in recent years to incorporate social aspects of waterfowl and habitat conservation in the North American Waterfowl Management Plan, through working groups, surveys, training programs, and other efforts (NAWMP 2018). Adjustments can also include periodic changes in the regulatory alternatives and the protocols for monitoring, and other elements of decision making (Johnson 2011).

In addition to adaptive harvest management of waterfowl, there are a number of other well-developed applications of adaptive management that could be used to illustrate a complete cycle of adaptive management. A notable example includes adaptively managing the commercial take of horseshoe crabs (*Limulus polyphemus*) in Delaware Bay each spring, where the eggs deposited by crabs serve as a critical food source for migrating red knots (*Calidris canutus rufa*) (McGowan et al. 2009; Smith et al. 2013; McGowan et al. 2015; Robinson et al. 2017). Another example involves the adaptive management of water flows on the Tallapoosa River to meet multiple biological, recreational, and energy objectives (Irwin and Freeman 2002). Moore and Conroy (2006) discuss the use of adaptive management in Southeastern pine forests in the United States for recovery of the endangered red-cockaded woodpecker (*Leuconotopicus borealis*). Martin et al. (2009, 2011) describe the adaptive management of human disturbance in Denali National Park to sustain golden eagle (*Aquila chrysaetos*) populations while permitting recreational use of the park. Adaptive management has also been used in managing endangered plants (Moore et al. 2011a), managing fire to enhance habitat for a threatened species, the Florida scrub jay (*Aphelocoma coerulescens*) (Johnson et al. 2011), managing logging and fire on old-growth forests in the Pacific Northwest (Noon and Blakesley 2006; Healey et al. 2008), managing water releases in the Columbia Basin to sustain Chinook salmon (*Oncorhynchus tshawytscha*) (Marcot et al. 2001), and a wide variety of other projects.

In the next sections, we provide examples of institutional learning in adaptive management, and also discuss some ways that uncertainties about the decision components can be reduced in the course of institutional learning.

## Institutional Learning

One consequence of the dynamism of natural resources management is the potential for the decision-making process to be adjusted as understanding grows or stakeholders’ preferences change. In this section, we consider potential mechanisms for evidence-based change in the decision elements of the deliberative phase as management proceeds.

### Management Objectives

A standard application of adaptive decision making assumes explicit and agreed-upon objectives for management. Yet it is not unusual for there to be uncertainty (or disagreement) about objectives, with different stakeholders expressing different views not only about system responses to management but also about which management objectives are most appropriate. In fact, individual stakeholders often recognize and value multiple objectives, and there typically are differences of opinion among stakeholders about the relative importance of these objectives (Norton 2005). In our waterfowl management example, two important programs for managing waterfowl in the United States—the federal regulation of harvest management and the North American Waterfowl Management Plan—have different objectives, in that federal regulation tends toward management for maximum harvest, whereas the North American Waterfowl Management Plan specifies target population sizes (Johnson 2011). An integration of adaptive harvest management and the North American Waterfowl Management Plan will ultimately require reconciliation and development of common management objectives (Runge et al. 2006; Johnson 2011).

The evolution of objectives in adaptive management requires their identification not only at the initiation of a process, but also at points going forward as decisions are made and evidence of performance is gathered. A common strategy is to engage in objective setting on the front end of a project in order to guide decision making and performance evaluation, and then to adjust objectives as needed when stakeholder preferences and perspectives change, decision making is evaluated, and learning increases. In the example from waterfowl adaptive harvest management, the codified objective has been to maximize long-term harvest, whereas managers today are more focused on objectives relating to hunter satisfaction and participation rather than the size of the harvest (Johnson et al. 2015). This example also shows that explicit recognition of social objectives (hunter satisfaction, participation) has become an important complement to biological objectives (maintaining population size, regulating harvest magnitude) in increasing understanding of waterfowl management. In another example, the original objective of alligator population management in Florida was avoiding the risk of population declines, but when alligator populations grew larger and led to more public complaints about nuisance alligators, the objective changed to keeping populations within 25% of 1980s levels (Tyre and Michaels 2011). A possible approach for considering different objectives is to articulate a range of alternative objectives at the beginning of a project, in anticipation of future adjustment.

Several ways to identify and weight objectives can be considered. One is to use a formal or informal survey to elicit stakeholders’ priorities for possible objectives, as described by Irwin and Kennedy (2008) for management of water releases from a river dam. A number of techniques are available that rely on expert judgment and other experience of biologists and analysts (Reed et al. 2009; Runge et al. 2011; Gregory et al. 2012). Other methods use the record of prior experience, perhaps relying on objectives and management actions in previous cycles of adaptive management. In the example from waterfowl management, decades of harvest management have provided an in-depth understanding of duck population dynamics, which can be used as a basis to redefine the harvest and habitat objectives of both the adaptive harvest management program and the North American Waterfowl Management Plan so that the two sets of objectives are concordant (Runge et al. 2006).

A particular technical procedure for adjusting objectives was given by Williams (2012), who extended the treatment of structural uncertainty to include uncertainty about the objectives. The general idea is to elicit an array of feasible objectives from stakeholders, and develop a composite objective with weights that evolve with the resource system’s responses to management. On the assumption that there is a stochastic linkage between the uncertainties about system structure and the management objectives, the assessment of data can be used to reduce both kinds of uncertainty. In such a context, objectives themselves can be treated as hypotheses to be examined with monitoring data, just as the system models can be thought of as hypotheses to be investigated with data. Thus, the stochastic linkage between models and objectives allows the use of monitoring data to update the weights for both the models and objectives (Williams 2012). The updating of objective weights represents learning about the objectives, in the same way that adjustment of model weights represents learning about the resource system.

Of the many approaches to the adaptive revision of objectives that can be envisioned, some are quite technical and others are less so. Which means to employ depends very much on the resource problem, the stakeholder community, and the level of available technical expertise.

### Alternative Management Actions

Potential management actions, as well as objectives, may be subject to change over time as a resource is managed. The revision of alternatives can be occasioned by many factors, such as shifts in stakeholders’ viewpoints, addition of new stakeholders, improved understanding of the system, or increased information about the effects of different management interventions. In our example of the regulatory management of waterfowl hunting, managers have changed the management alternatives, i.e., the set of permissible regulatory frameworks of hunting season dates and bag limits, in an effort to increase hunting opportunities and enhance hunters’ satisfaction and participation (Johnson 2011).

Revision of the management alternatives can take into account both the completeness of the set of alternatives as well as variation in the impacts of alternatives. The identification of an effective strategy depends upon contrasts among the predicted outcomes of management actions, in that contrasting outcomes enable one to recognize their relative management value (Williams and Brown 2016). Conversely, adaptive decision making can be compromised if the potential actions produce essentially indistinguishable predictions.

Several factors can suggest the need to change the set of alternatives. For example, an initially agreed-upon set of management alternatives may include options with little difference in their predicted impacts; or one or more of the potential actions under consideration may never be selected, because more valuable alternatives can always be found; or it may become clear through multiple iterations of decision making that feasible alternatives were overlooked in the original set of actions. In such instances, it is important to consider revising the set of management alternatives.

An outcome-based approach is often used to add or eliminate actions. In our waterfowl example, changes in regulatory options have been made several times, and have included both expansion and reduction of the number of options, as understanding of waterfowl populations has improved and stakeholder perceptions have changed (Johnson 2006; Conroy et al. 2011). Another example is given by Ascoli et al. (2009), who designed an active adaptive management project for experimental learning about the most effective management (prescribed fire, grazing, cutting) for preventing overgrowth of European heathlands. Monitoring of ecological impacts of each alternative action was used to discover and discard the actions that were ineffective for heathland conservation. A third example focuses on the effectiveness of management actions (supplemental feeding, mite control) for increasing vital rates in a translocated population of the hihi (*Notomystis cincta*), an endangered New Zealand forest bird (Armstrong et al. 2007), with the elimination of management treatments that did not increase female productivity. An example involving the inclusion of new management actions is presented by Perkins et al. (2011), who described new actions (delayed mowing of pastures, crop rotations) incorporated in Scottish agricultural land-use schemes when corn bunting (*Emberiza calandra*) populations were shown to increase.

Various criteria for changing alternatives can be used. One criterion could be the failure of an option to be chosen after many iterations of decision making. Analytic methods might involve tracking the likelihood of an alternative being selected. The conditions under which an alternative would be selected can also be analyzed, along with the likelihood of occurrence of those conditions. Finally, the collective experience of stakeholders in managing a resource can motivate reconsideration of the set of management actions. In one such example, agricultural stakeholders suggested a new set of management actions for the second iteration of an adaptive management project to mitigate risk of the water-borne pathogen *Cryptosporidium* in a rural Australian catchment (Bryan et al. 2009). Scenario analysis was then used to ascertain which of the new actions (e.g., restricting calves’ access to watercourses) was most likely to be effective in achieving further reduction of *Cryptosporidium* in the forthcoming project cycle.

### Models

The set of predictive models expressing different hypotheses about the resource system’s dynamics and responses to management may also be modified over time, as stakeholders’ viewpoints change and new understanding develops. In our example of waterfowl hunting regulations, population dynamics resulting from previously unobserved environmental conditions have suggested that current models of system dynamics may be inadequate (Johnson 2011). Similarly, social elements and changes in the human dimensions of waterfowl management that were clearly recognized in the 2012 revision of the North American Waterfowl Management Plan (NAWMP 2018) are also important components of system models.

Identifying effective strategies and promoting learning depends not only on variation among predicted impacts of alternative actions, but also on variation in performance among models. That is, models incorporating different hypotheses about how the resource system works should generally produce distinctly different predictions about the impacts of a given management action. There is little practical value in resolving uncertainty about how a system works if the models describing the system predict identical outcomes.

The parallels between models and management alternatives in adaptive management suggest that they should be considered in combination. That is, adaptive decision making works best when (1) there is substantial variation in the hypotheses about structure and dynamics of the resource system, i.e., in the models representing the system; and (2) there is substantial variation in the management alternatives with regard to their predicted impacts on the resource system (Williams and Brown 2016)

There are several avenues for reviewing models. One way is to address redundancy in the model set by comparing each model’s predicted responses to the alternative actions. If two or more models predict nearly identical responses by the system, only one of them is needed. Even if the models generate different predictions about the system’s responses, the inclusion of both will not be needed for management if they both lead to the same strategy and values.

Another method might be to investigate the record of adaptive decision making to determine the usefulness of particular models. A rapid and consistent decline in the weight (and hence, confidence) associated with a model is indicative of a mismatch between the model’s predictions and the actual data produced by monitoring the system, suggesting the model’s inadequacy in representing system dynamics and predicting future system behavior. For example, Pine et al. (2009) reviewed numerous efforts in fisheries to improve or restore fishery performance or improve habitat, with results frequently indicating that models made incorrect predictions about how an ecosystem would respond, and thus providing an opportunity for model improvement or replacement.

Finally, it may be that the model set as a whole is inadequate, in that none of the models are especially effective in representing system dynamics. This is evidenced by an ongoing mismatch of model projections in comparison with observed data, which suggests that an appropriate model of the resource system is not included in the model set. Under these circumstances, it is useful to consider developing other models. In the waterfowl example, all of the current models of waterfowl population dynamics used in adaptive harvest management, which are based on the assumption that climate processes are stationary, may need to be redeveloped to incorporate climate change–induced nonstationarity of wetland dynamics (Johnson et al. 2015). In another example, when grassland restoration using state-and-transition models based on prevailing understanding of fire thresholds in juniper woodland was unsuccessful (Twidwell et al. 2013a), refinement of the models with data from fire physics produced new quantitative estimates of the fire intensity needed to kill encroaching junipers, and resulted in successful restoration after increased use of high-intensity fires (Twidwell et al. 2013a). In a further example, Cummings et al. (2005) applied adaptive experimentation for restoration of derelict sand mining sites, and used projected restoration outcomes (seedling survival, weed proliferation) to assess the need to include other system features (herbivore browsing, soil deficiencies) to improve restoration strategies.

Outstanding issues in adaptive revision of the model set are how to measure the inadequacy of the existing models, how to identify a critical level of that inadequacy, and how to select and weight new models. Some procedures for determining the models in multi-model inference techniques (Rehme et al. 2011) may provide a starting point for analysis. Another technique has been suggested by Runge et al. (2016), who used a case example of greater-than-expected Arctic sea ice loss to show that empirical distribution function tests for examining the agreement between two continuous functions can be used to identify whether the current model ensemble is plausible, i.e., is bounding the behavior of the system.

### Monitoring

Monitoring plays a critical role in adaptive management, by producing data with which to estimate resource status, inform decision making, and facilitate evaluation and learning after decisions are made. Monitoring is an ongoing activity that is conducted according to protocols developed in the deliberative phase of adaptive management, and is not simply after-the-fact tracking in the absence of any capacity to contrast actual results with predicted responses (Nichols and Williams 2006). In our example of the regulation of waterfowl hunting, monitoring focuses on waterfowl population status and wetland conditions on the breeding grounds. A revision of the models in adaptive harvest management to include new population attributes or environmental conditions could necessitate a corresponding revision in monitoring programs and protocols to target the newly identified features.

There are several issues that are relevant to the revision of monitoring protocols. One is the focus of monitoring. Over time, important but unmonitored system features may become apparent, or some monitored attributes may be found to have marginal value. Monitoring protocols may need to be adapted to this new understanding, so as to ensure the effectiveness of the monitoring effort. In the waterfowl hunting example, modifications of protocols due to new findings over time have affected the intensity, geographic coverage, and methodology of monitoring waterfowl abundance and distribution (Conroy et al. 2011; Johnson et al. 2015). In the same example, the increased focus on social objectives such as hunter satisfaction and participation could also occasion revision of monitoring programs to track these aspects, along with new research necessary to identify the most appropriate performance metrics (Johnson et al. 2015). In another example, Armstrong et al. (2007) reported that the monitoring of a re-introduced New Zealand forest bird, the hihi, led to a discovery of previously unrecognized mortality factors (nest mites, fungal spores), resulting in revised management actions and monitoring protocols. Marcot et al. (2012) documented the review of monitoring metrics used by the USDA Forest Service at Tongass National Forest on a regular schedule and their revision as needed to reflect new topics of social, economic, or scientific interest.

The evolving tradeoff between the cost and precision of monitoring also can motivate revisions in monitoring design. Advances in technology, changes in the spatial extent of the monitoring effort, increases or decreases in per-unit monitoring costs, and the perceived value of having more (or less) precision, can lead to a reconsideration of monitoring protocols.

A further consideration is the frequency of monitoring. Typically, adaptive decision making will be followed by monitoring, with the results used to inform decision making at the next decision event. However, it is certainly possible for the cadence of decision making to differ from that of monitoring (Williams and Johnson 2017), for example, if a management decision (such as seeding or prescribed burning in an ecological restoration project) requires a long planning period before it can be acted upon (Moore et al. 2011b). The potential savings in costs and time are important issues in establishing the frequencies of monitoring and decision making. Comparative valuations associated with different frequencies can highlight changes in precision and possible cost savings associated with less frequent monitoring (Williams and Johnson 2017). For example, explicit consideration of the costs and outcomes of monitoring within a decision-theoretic framework showed that the optimal level of monitoring of kangaroo populations in South Australia depended on the current state of the system, and in particular on whether the population was near a critical threshold of abundance (Hauser et al. 2006). A basic decision tree such as that proposed by McDonald-Madden et al. (2010) may also provide a helpful framework for evaluating monitoring costs and benefits in the context of a project’s financial and conservation issues.

Finally, the perceived value of monitoring by stakeholders can be altered by changing attitudes or priorities. Monitoring is often one of the most time-consuming and expensive aspects of adaptive management, and there is always a threat that it will be reduced or eliminated. Because of the critical role monitoring plays in adaptive management, dedicated resources and stakeholder commitments are crucial. When and how to modify monitoring protocols so as to retain stakeholders’ support while maintaining monitoring effectiveness is a critical consideration.

### Stakeholders

Within limits, revisitation and possible modification of the decision elements discussed thus far may be amenable to a technical assessment. The same is not necessarily the case for stakeholder engagement, even though learning about institutional arrangements and governance is the main result reported by the preponderance of adaptive management projects in practice (Fabricius and Cundill 2014). The participation of stakeholders is both a critical and highly complex component that underlies all aspects of adaptive decision making. In the waterfowl example, a full integration of two separate efforts, adaptive harvest management and the North American Waterfowl Management Plan, will require input from a much wider array of stakeholders, including informal networks of various public and private actors (Johnson 2011) as well as state and federal government managers, in order to foster the institutional change needed for new modes of resource governance. This challenge is especially relevant to the North American Waterfowl Management Plan, because it is a tri-national agreement involving many formal and informal networks of stakeholders in three countries.

In assessing stakeholder involvement, many attributes can be considered. The involvement of an appropriate number and mixture of stakeholders is essential, as is a governance structure such as a stakeholders’ board (Kennedy et al. 2007) or a multi-jurisdictional organization (Bischoff-Mattson and Lynch 2017) to ensure their input in the decision process. As always, effective communication is key, along with the commitment by stakeholders of the necessary time and resources (Kennedy et al. 2007; Irwin and Kennedy 2008; Moore et al. 2011b). In addition, a framework for interaction that allows for differences in perspectives, priorities, or preferences is required, as well as a process for reducing uncertainties and/or disagreements about management strategy. For example, in adaptively managing a riverine dam in the southeastern United States, stakeholders interacted with scientific experts and professional facilitators who guided the use of a Bayesian network decision support model in a series of workshops (Kennedy et al. 2007).

Robust participation of stakeholders is a key to success not only at the start of an adaptive management project, but consistently throughout the life of the project. Fujitani et al. (2017) found that active participation in adaptive management (of fish stocking) increased stakeholders’ knowledge and capacity for management of their fisheries resources. In a project for Great Plains grassland restoration via controlled burning (Twidwell et al 2013b), maintaining involvement of landowners’ groups has been a primary goal of monitoring and evaluating effectiveness of management actions (Allen et al. 2017). It is of course important to recognize that such meaningful stakeholder engagement involves increased costs to stakeholders in terms of the commitment of time and effort to attend meetings, develop knowledge, or represent a stakeholder group (Beckley et al. 2005).

When participation is strong at the beginning of a project but tapers off as other priorities intervene, periodic review and consideration of some or all of the foregoing stakeholder issues is necessary. The challenge is to design evidence-based criteria and identify mechanisms for their use in evaluating and adapting engagement approaches. Such a challenge will be specific to the resource problem and the particular stakeholder community.

## Discussion

With the maturation of adaptive management, emphasis on double-loop learning has steadily increased. There is by now a general recognition that as important as technical learning about the dynamics of resource systems is, learning about the elements of the decision-making architecture is equally important. The trend toward a more inclusive learning framework, along with a natural tendency for decision elements to evolve in conjunction with greater understanding and changing stakeholder priorities and perspectives, underscores the need for a more systematic approach to double-loop learning. Elements of such an approach include ways to recognize when the elements of decision making should be revisited, which elements should be adjusted, and how alternatives can be identified and incorporated on the basis of experience and management performance.

The challenges in designing a systematic approach to institutional learning are greater than those for technical learning. The focus of technical learning is on the reduction of uncertainty about the structure and dynamics of the resource system, as represented by a set of predictive models and confidence weights for them. Mechanisms such as hierarchical modeling (Royle and Dorazio 2008) and Bayesian inference (Link and Barker 2010; Hobbs and Hooten 2015) are available to determine the rate and direction of learning over time. However, it is not so clear how to measure institutional uncertainty, how to propagate it over time, and how to identify evidence-based adjustments of the elements of decision making.

In the foregoing sections of this article, we have described a framework and suggested methodology for institutional learning that complement that for technical learning. It should be emphasized that not all elements of the decision-making cycle are equally amenable to this framework. A stochastic linkage between structural uncertainty and the uncertainty related to management objectives allows for a treatment that includes metrics, the propagation of uncertainty, and mechanisms for changing objectives over time. Yet such formal methods are not obviously suited for engaging stakeholders, not least because of the large number of factors involved in tracking stakeholder engagement and evaluating the need and potential for change.

In general, the challenges associated with institutional learning can be handled more directly to the extent that the methods of technical learning can be adapted to the decision elements. For example, revision of the set of alternative management actions can be addressed with measures based on management performance, such as the frequency with which a particular alternative action is actually chosen and the potential for its selection, as well as projections of management improvement with the inclusion of new alternatives. Similarly, revision of the set of predictive models can be addressed with metrics tied to management performance, for example, the pattern of weighting of a particular model as an indicator of model adequacy in representing resource system dynamics. One can also conduct comparative assessments of multiple models to explore redundancies in their performance.

On the other hand, it is much less evident how to identify the metrics for monitoring. A good starting point is to consider relevance, cost, and precision. Because the role of monitoring is critical in adaptive management, it is obviously important that monitoring protocols focus on system features and attributes that are subject to uncertainty, and to modify protocols as needed to improve that focus. One can track monitoring costs and make alterations as per-unit costs or budgets for monitoring change. Attention should also be given to changes in technology and other factors that can affect precision.

Similarly, tracking stakeholder engagement and participation is not as clear-cut as evaluating technical performance. There is a wide array of tools for participation, and multiple tools are likely to be needed for successful stakeholder participation in any given project (Beckley et al. 2005). For example, “indirect” methods such as surveys or polling are good for gathering representative values on general issues, whereas “direct” methods such as advisory boards or workshops are better for establishing dialogue and identifying workable compromises (Beckley et al. 2005). Beckley et al. (2005) identified three criteria (breadth, depth, outcomes) to measure success. Indicators of increased knowledge or relevance to decision making are often as important to evaluate as simple procedural indicators of participation (e.g., frequency of meetings), although more difficult. Well-organized stakeholder participation can be very time consuming and require specialized social science techniques, but ultimately result in better decisions (Beckley et al. 2005).

In general, a change in thinking about the respective roles of technical and institutional learning will require cultural as well as operational changes. Institutions are built on major premises and long-held beliefs that are deeply embedded in educational systems, laws, policies, and norms of professional behavior (Miller 1999). There is a natural tension between the tendency to maintain a strong institutional framework for thinking and decision making, versus adaptive decision making that relies on awareness of alternative perspectives, acceptance of uncertainty, and especially collaboration and flexibility (Gunderson 1999). A strong predisposition for the status quo works to the detriment of institutional learning and adjustment of the decision cycle.

Nevertheless, we believe that there are real benefits in expanding the framework of technical learning in adaptive management to allow for adaptation of the architecture of the decision making. Perhaps the greatest benefit is in promoting the continuing support and involvement of stakeholders, because the role stakeholders play is critical in all aspects of adaptive decision making. Flexible, evidence-based adaptation, including institutional adaptation, can contribute to broader participation, greater enthusiasm, and better and less contentious resource management.
